# Author Correction: Child wasting and concurrent stunting in low- and middle-income countries

**DOI:** 10.1038/s41586-023-06695-0

**Published:** 2023-10-13

**Authors:** Andrew Mertens, Jade Benjamin-Chung, John M. Colford, Alan E. Hubbard, Mark J. van der Laan, Jeremy Coyle, Oleg Sofrygin, Wilson Cai, Wendy Jilek, Sonali Rosete, Anna Nguyen, Nolan N. Pokpongkiat, Stephanie Djajadi, Anmol Seth, Esther Jung, Esther O. Chung, Ivana Malenica, Nima Hejazi, Haodong Li, Ryan Hafen, Vishak Subramoney, Jonas Häggström, Thea Norman, Parul Christian, Kenneth H. Brown, Benjamin F. Arnold, The Ki Child Growth Consortium, Tahmeed Ahmed, Asad Ali, France Begín, Pascal Obong Bessong, Zulfiqar A. Bhutta, Robert E. Black, Ladaporn Bodhidatta, William Checkley, Jean E. Crabtree, Rina Das, Subhasish Das, Christopher P. Duggan, Abu Syed Golam Faruque, Wafaie W. Fawzi, José Quirino da Silva Filho, Robert H. Gilman, Richard L. Guerrant, Rashidul Haque, Eric R. Houpt, Najeeha Talat Iqbal, Jacob John, Sushil Matthew John, Gagandeep Kang, Margaret Kosek, Aldo Ângelo Moreira Lima, Tjale Cloupas Mahopo, Dharma S. Manandhar, Karim P. Manji, Estomih Mduma, Venkata Raghava Mohan, Sophie E. Moore, Mzwakhe Emanuel Nyathi, Maribel Paredes Olortegui, William A. Petri, Prasanna Samuel Premkumar, Andrew M. Prentice, Najeeb Rahman, Kamran Sadiq, Rajiv Sarkar, Naomi M. Saville, Bhim P. Shrestha, Sanjaya Kumar Shrestha, Bakary Sonko, Erling Svensen, Sana Syed, Fayaz Umrani, Honorine D. Ward, Pablo Penataro Yori

**Affiliations:** 1 Division of Epidemiology and Biostatistics, University of California, Berkeley, Berkeley, CA USA; 2 Department of Epidemiology and Population Health, Stanford University, Stanford, CA USA; 3 Chan Zuckerberg Biohub, San Francisco, CA USA; 4 Hafen Consulting, West Richland, WA USA; 5 DVPL Tech, Dubai, United Arab Emirates; 6 Cytel, Waltham, MA USA; 7 Quantitative Sciences, Bill & Melinda Gates Foundation, Seattle, WA USA; 8 Center for Human Nutrition, Department of International Health, Johns Hopkins Bloomberg School of Public Health, Baltimore, MD USA; 9 Department of Nutrition, University of California, Davis, Davis, CA USA; 10 Francis I. Proctor Foundation, University of California, San Francisco, CA USA; 11 Department of Ophthalmology, University of California, San Francisco, CA USA; 12 International Centre for Diarrhoeal Disease Research, Dhaka, Bangladesh; 13 Department of Pediatrics and Child Health, Aga Khan University, Karachi, Pakistan; 14 UNICEF, New York, NY USA; 15 HIV/AIDS and Global Health Research Programme, University of Venda, Thohoyandou, South Africa; 16 Centre of Excellence in Women and Child Health, Institute for Global Health and Development, The Aga Khan University, Karachi, Pakistan; 17 Johns Hopkins University Bloomberg School of Public Health, Baltimore, MD USA; 18 Armed Forces Research Institute of Medical Sciences, Bangkok, Thailand; 19 Leeds Institute for Medical Research, St James’s University Hospital, University of Leeds, Leeds, UK; 20 Center for Nutrition, Boston Children’s Hospital, Boston, MA USA; 21 Department of Global Health and Population, Harvard T.H. Chan School of Public Health, Boston, MA USA; 22 Federal University of Ceará, Fortaleza, Brazil; 23 University of Virginia, Charlottesville, VA USA; 24 Christian Medical College, Vellore, India; 25 Low Cost Effective Care Unit, Christian Medical College, Vellore, India; 26 Translational Health Science and Technology Institute, Faridabad, India; 27 Department of Nutrition, School of Health Sciences, University of Venda, Thohoyandou, South Africa; 28 Mother and Infant Research Activities, Kathmandu, Nepal; 29 Department of Pediatrics and Child Health, Muhimbili University School of Health and Allied Sciences, Dar es Salaam, Tanzania; 30 Haydom Lutheran Hospital, Haydom, Tanzania; 31 Department of Women and Children’s Health, Kings College London, London, UK; 32 MRC Unit The Gambia at London School of Hygiene and Tropical Medicine, Banjul, The Gambia; 33 Department of Animal Sciences, School of Agriculture, University of Venda, Thohoyandou, South Africa; 34 AB PRISMA, Lima, Peru; 35 Institute for Global Health, University College London, London, UK; 36 Health Research and Development Forum, Kathmandu, Nepal; 37 Walter Reed AFRIMS Research Unit, Kathmandu, Nepal; 38 Haukeland University Hospital, Bergen, Norway; 39 Tufts Medical Center, Tufts University School of Medicine, Boston, MA USA

**Keywords:** Malnutrition, Paediatric research, Risk factors, Developing world, Epidemiology

Correction to: *Nature* 10.1038/s41586-023-06480-z Published online 13 September 2023

In the version of the article initially published, the author list for The Ki Child Growth Consortium was incorrect. Rina Das, Subhasish Das and Najeeb Rahman have been added to The Ki Child Growth Consortium author list, while Souheila Abbeddou, Linda S. Adair, Hasmot Ali, Per Ashorn, Rajiv Bahl, Mauricio L. Barreto, Maharaj Kishan Bhan, Nita Bhandari, Santosh K. Bhargava, Delia Carba, Ines Gonzales Casanova, Kathryn G. Dewey, Caroline H. D. Fall, Sonja Y. Hess, Jean H. Humphrey, Elizabeth Yakes Jimenez, Michael S. Kramer, Alain Labrique, Nanette R. Lee, Mustafa Mahfuz, Kenneth Maleta, Reynaldo Martorell, Sarmila Mazumder, Ishita Mostafa, Robert Ntozini, Harshpal Singh Sachdev, Saijuddin Shaikh, Alberto Melo Soares, Aryeh D. Stein, Keith P. West Jr, Lee Shu Fune Wu and Seungmi Yang have been removed. This has been updated in the PDF and HTML versions of the article.

