# Supplementary material for: Child wasting and concurrent stunting in low- and middle-income countries
Source: Nature. 2023 Sep 13;621(7979):558–67. doi: 10.1038/s41586-023-06480-z (PMC10511327; doi:10.1038/s41586-023-06480-z)

nature portfolio

https://doi.org/10.1038/s41586-023-06480-z

Supplementary information

Child wasting and concurrent stunting in low- and middle-income countries

In the format provided by the authors and unedited

Supplementary Information for:

Child wasting and concurrent stunting in low- and middle-income countries

Andrew Mertens,[1]* Jade Benjamin-Chung,[2,3,4] John M Colford Jr,[1] Alan E Hubbard,[1] Mark J van der Laan,[1] Jeremy Coyle[1], Oleg Sofrygin,[1] Wilson Cai,[1] Wendy Jilek,[1] Sonali Rosete,[1] Anna Nguyen,[1] Nolan N Pokpongkiat,[1] Stephanie Djajadi,[1] Anmol Seth,[1] Esther Jung,[1] Esther O Chung,[1] Ivana Malenica,[1] Nima Hejazi,[1] Haodong Li,[1] Ryan Hafen,[5] Vishak Subramoney,[6] Jonas Häggström,[7] Thea Norman,[8] Parul Christian,[9] Kenneth H Brown,[10] Benjamin F. Arnold,[11] and members of the *ki* Child Growth Consortium

[1] Division of Epidemiology & Biostatistics, University of California, Berkeley, 2121 Berkeley Way Rm 5302 Berkeley, CA 94720-7360

[2] Department of Epidemiology & Population Health, Stanford University, Stanford University, 300 Pasteur Dr., Stanford, CA 94305

[3] Division of Epidemiology & Biostatistics, University of California, Berkeley, 2121 Berkeley Way Rm 5302 Berkeley, CA 94720-7360

[4] Chan Zuckerberg Biohub, San Francisco, CA 94158

[5] Hafen Consulting, LLC, West Richland WA, 99353

[6] DVPL Tech

[7] Cytel Inc, 1050 Winter St Suite 2700 Waltham, MA 02451, USA

[8] Quantitative Sciences, Bill & Melinda Gates Foundation, 500 5th Ave N, Seattle, WA 98109

[9] Center for Human Nutrition, Department of International Health, Johns Hopkins Bloomberg School of Public Health, Baltimore, MD 21205.

[10] Department of Nutrition, University of California, Davis, 3135 Meyer Hall, Davis, CA 95616-5270

[11] Francis I. Proctor Foundation and Department of Ophthalmology, University of California, San Francisco, 490 Illinois St, San Francisco, CA 94158

Corresponding authors:

Andrew Mertens, PhD, amertens@berkeley.edu, Research Data Analyst, Division of Epidemiology & Biostatistics, University of California, Berkeley, 2121 Berkeley Way Rm 5302 Berkeley, CA 94720-7360

Benjamin F. Arnold, PhD, ben.arnold@ucsf.edu, Associate Professor, Francis I. Proctor Foundation, University of California, San Francisco, 490 Illinois St, San Francisco, CA 94158

Supplementary Note 1. Anthropometry measurement quality

1.1 Anthropometry measurements compared to WHO standards

To check for outliers in length measurements, we plotted the distribution of raw length and weight measurements by age and sex against bands marking the first, second, and third standard deviations of the World Health Organization child growth standard distribution. The majority of observations fell within 3 standard deviations of the mean of the standard for males and females.

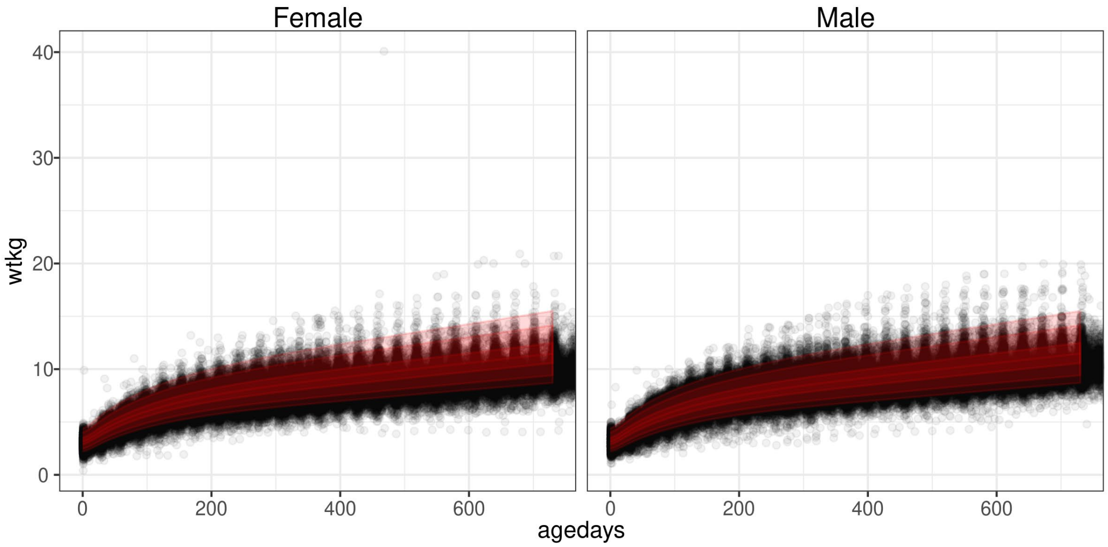
Female
Male
wtkg
agedays
0
10
20
30
40
0
200
400
600

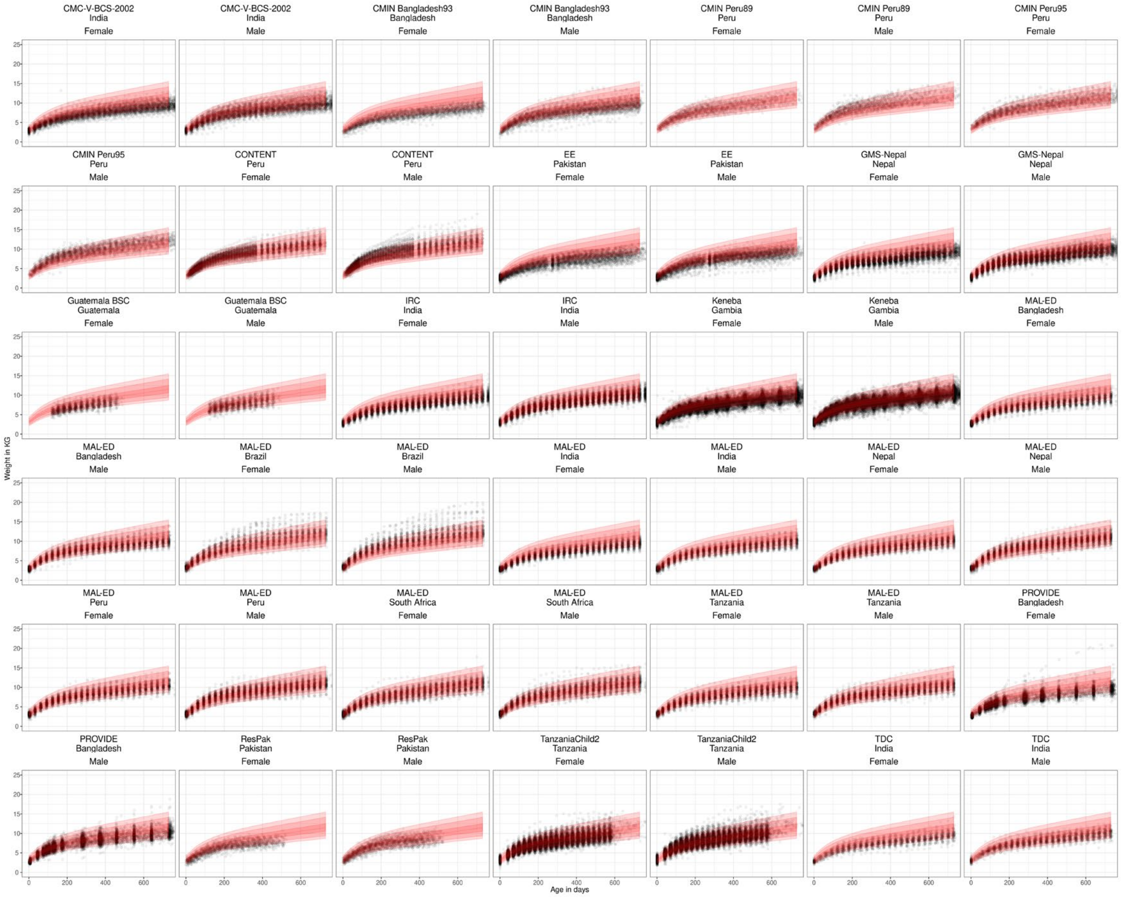
CMC-V-BCS-2002 India Female
CMC-V-BCS-2002 India Male
CMIN Bangladesh93 Bangladesh Female
CMIN Bangladesh93 Bangladesh Male
CMIN Peru89 Peru Female
CMIN Peru89 Peru Male
CMIN Peru95 Peru Female
CMIN Peru95 Peru Male
CONTENT Peru Female
CONTENT Peru Male
EE Pakistan Female
EE Pakistan Male
GMS-Nepal Nepal Female
GMS-Nepal Nepal Male
Guatemala BSC Guatemala Female
Guatemala BSC Guatemala Male
IRC India Female
IRC India Male
Keneba Gambia Female
Keneba Gambia Male
MAL-ED Bangladesh Female
MAL-ED Bangladesh Male
MAL-ED Brazil Female
MAL-ED Brazil Male
MAL-ED India Female
MAL-ED India Male
MAL-ED Nepal Female
MAL-ED Nepal Male
MAL-ED Peru Female
MAL-ED Peru Male
MAL-ED South Africa Female
MAL-ED South Africa Male
MAL-ED Tanzania Female
MAL-ED Tanzania Male
PROVIDE Bangladesh Female
PROVIDE Bangladesh Male
ResPak Pakistan Female
ResPak Pakistan Male
TanzaniaChild2 Tanzania Female
TanzaniaChild2 Tanzania Male
TDC India Female
TDC India Male
Weight in KG
Age in days

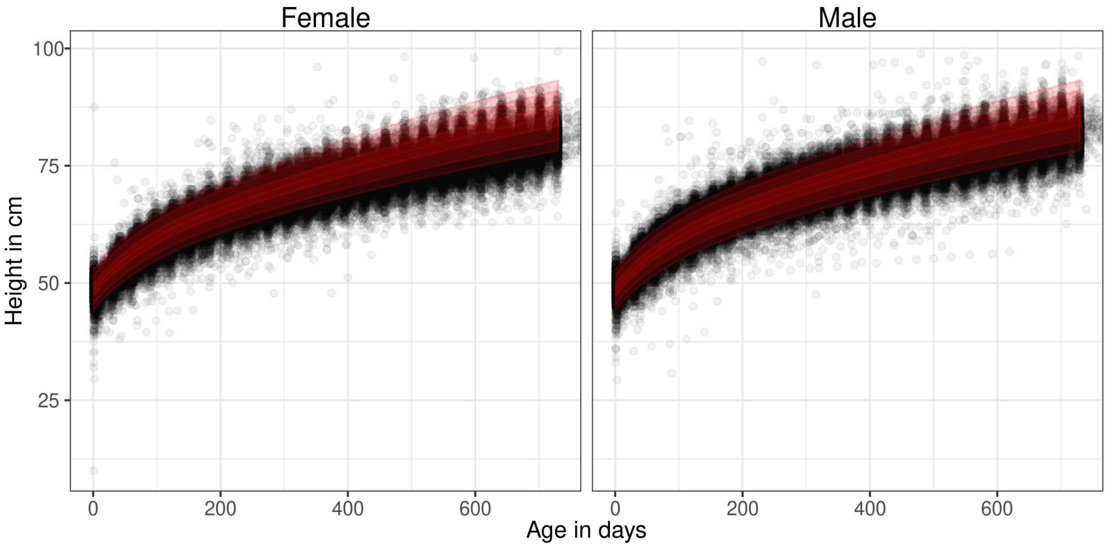
Female
Male
Height in cm
100
75
50
25
0
200
400
600
Age in days

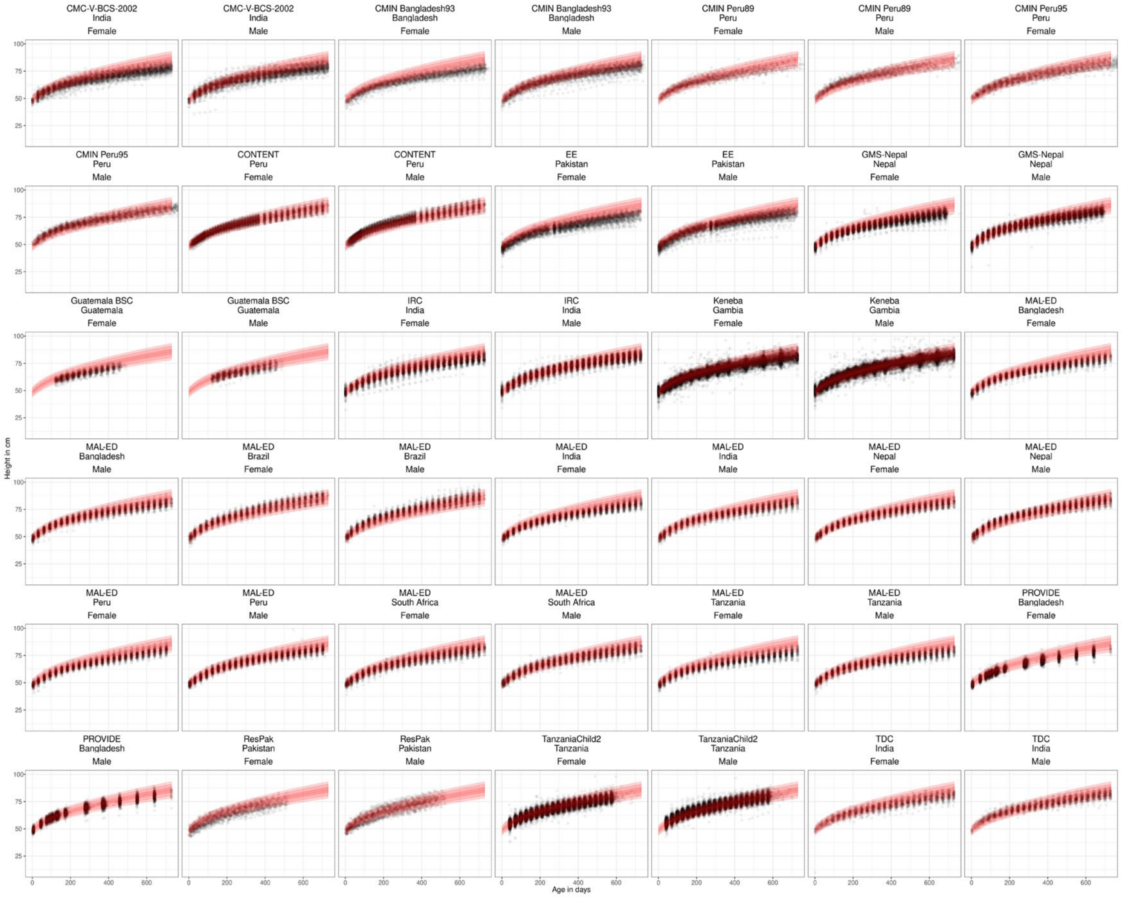
CMC-V-BCS-2002 India Female
CMC-V-BCS-2002 India Male
CMIN Bangladesh93 Bangladesh Female
CMIN Bangladesh93 Bangladesh Male
CMIN Peru89 Peru Female
CMIN Peru89 Peru Male
CMIN Peru95 Peru Female
CMIN Peru95 Peru Male
CONTENT Peru Female
CONTENT Peru Male
EE Pakistan Female
EE Pakistan Male
GMS-Nepal Nepal Female
GMS-Nepal Nepal Male
Guatemala BSC Guatemala Female
Guatemala BSC Guatemala Male
IRC India Female
IRC India Male
Keneba Gambia Female
Keneba Gambia Male
MAL-ED Bangladesh Female
MAL-ED Bangladesh Male
MAL-ED Brazil Female
MAL-ED Brazil Male
MAL-ED India Female
MAL-ED India Male
MAL-ED Nepal Female
MAL-ED Nepal Male
MAL-ED Peru Female
MAL-ED Peru Male
MAL-ED South Africa Female
MAL-ED South Africa Male
MAL-ED Tanzania Female
MAL-ED Tanzania Male
PROVIDE Bangladesh Female
PROVIDE Bangladesh Male
ResPak Pakistan Female
ResPak Pakistan Male
TanzaniaChild2 Tanzania Female
TanzaniaChild2 Tanzania Male
TDC India Female
TDC India Male
Height in cm
Age in days

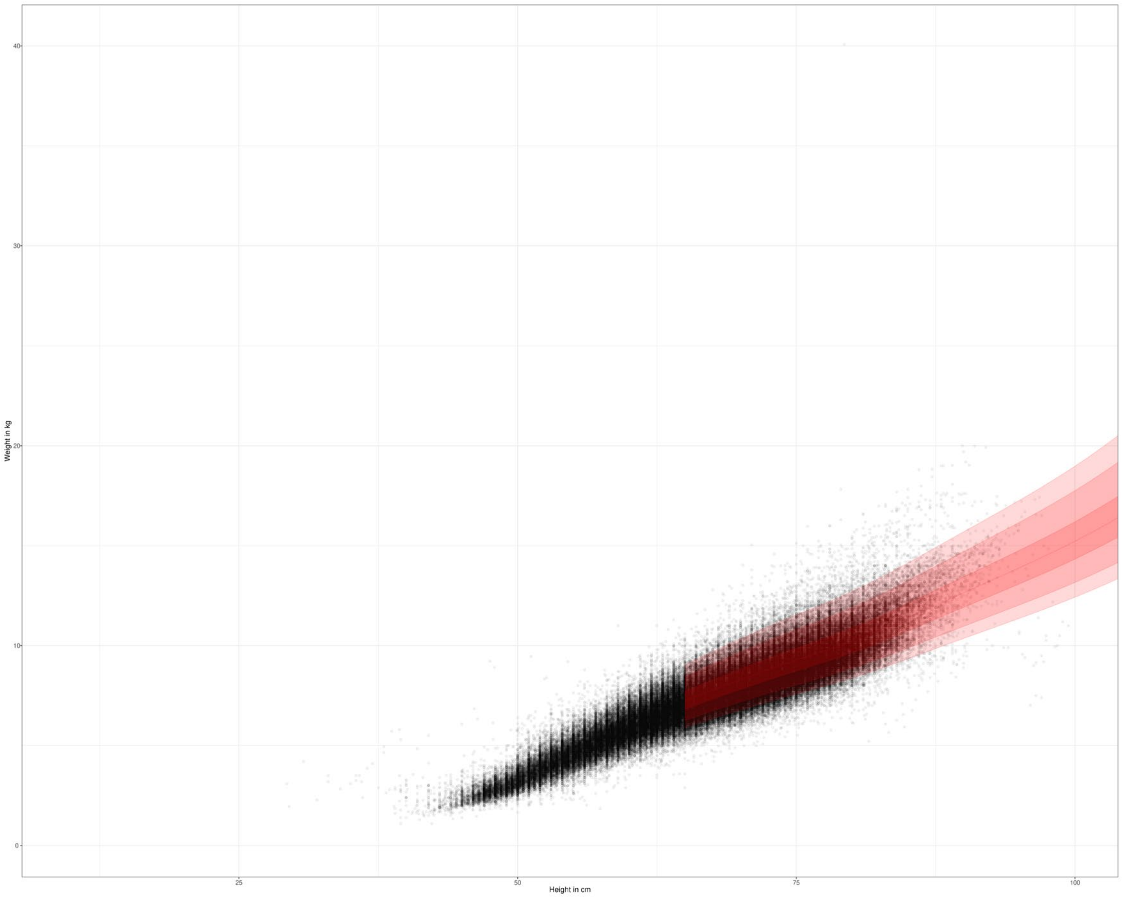

Weight in kg
Height in cm
0
10
20
30
40
25
50
75
100

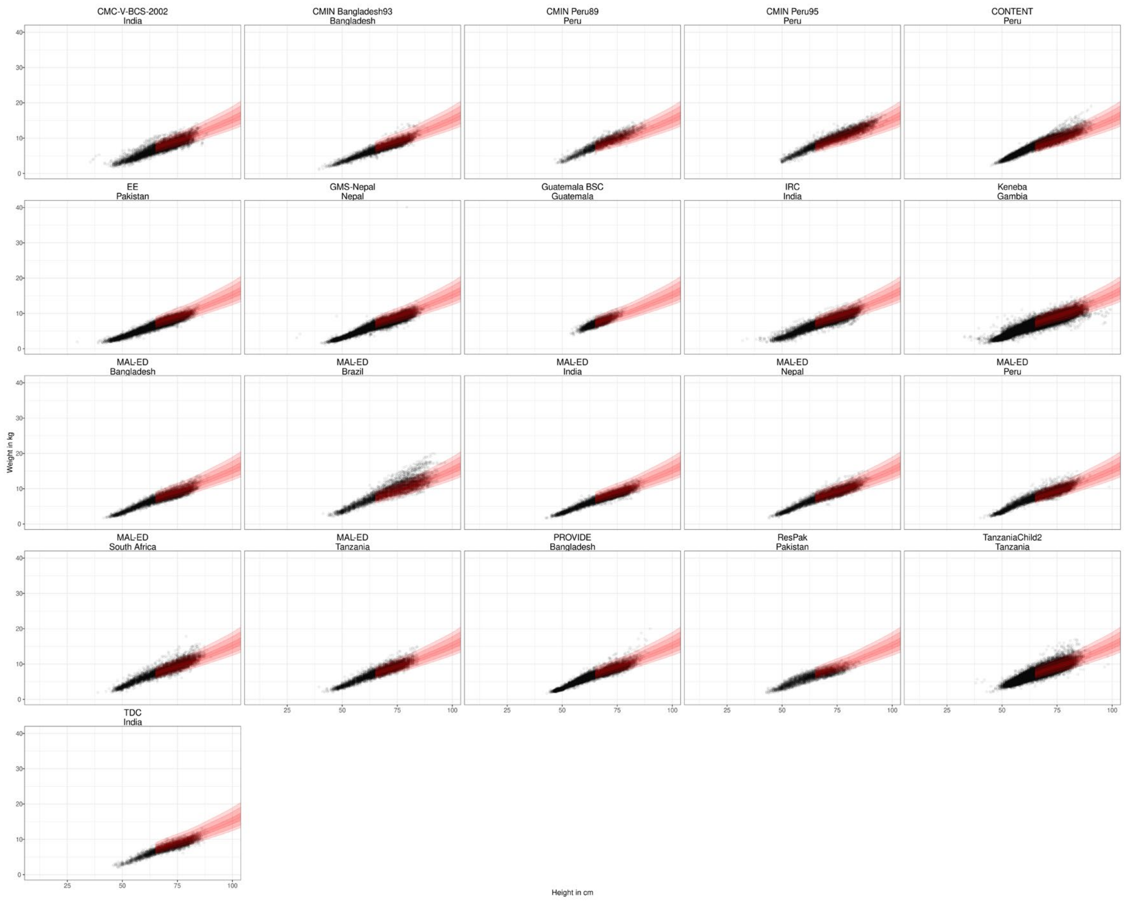

1.2 Age-specific incidence

This study included cohorts that measured child growth from 1987 to 2014. To assess potential secular trends, we plotted the mean LAZ, WAZ, and WLZ over time. The plot below shows the individual observations from included studies over this range of years. There does not appear to be a secular trend in LAZ, WAZ, or WLZ.

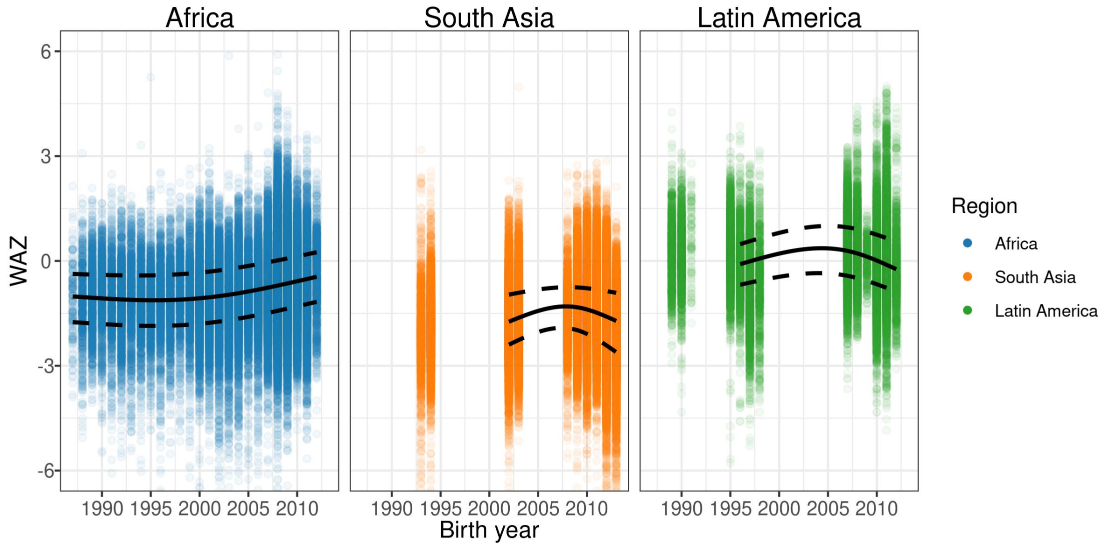

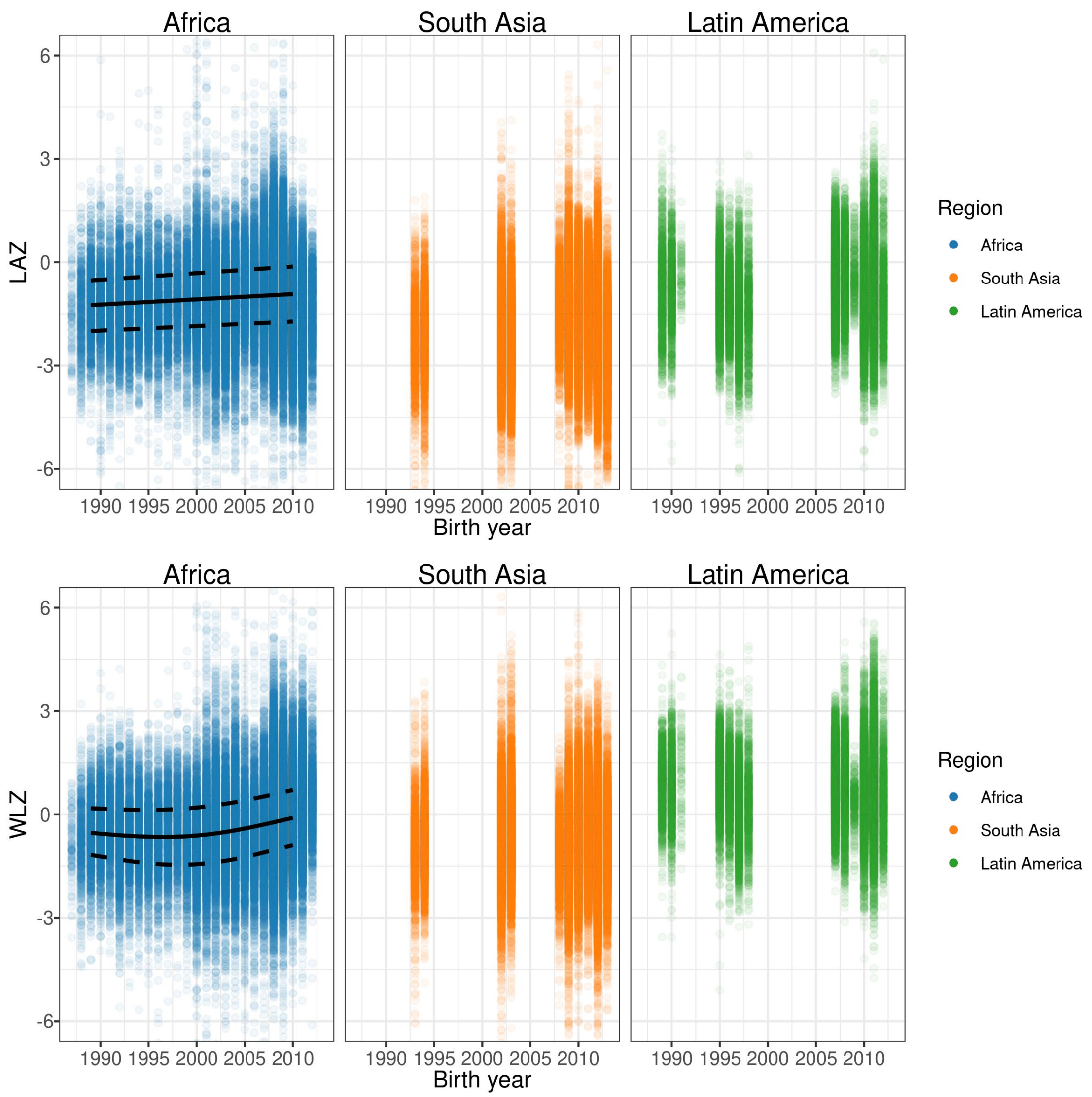

Supplementary Note 2. Sensitivity analysis using fixed effects

2.1 Overview

The primary analyses presented in this manuscript pooled across individual studies using random effects. Inferences about estimates from fixed effects models are restricted to only the included studies.[1] The random effects approach is more conservative in the presence of study heterogeneity and has larger confidence intervals around each point estimate unless all cohort-specific estimates are very similiar. Overall, the inference from results produced by each method did not greatly differ.

---

[1] Hedges, L. V. & Vevea, J. L. Fixed- and random-effects models in meta-analysis. Psychol. Methods 3, 486–504 (1998).

2.2 Age-specific prevalence

2.2.1 Random effects

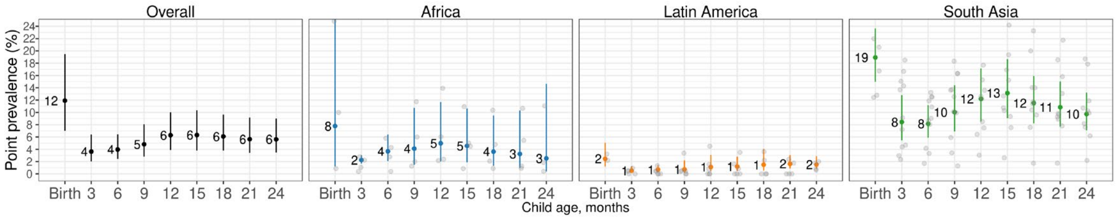

2.2.2 Fixed effects

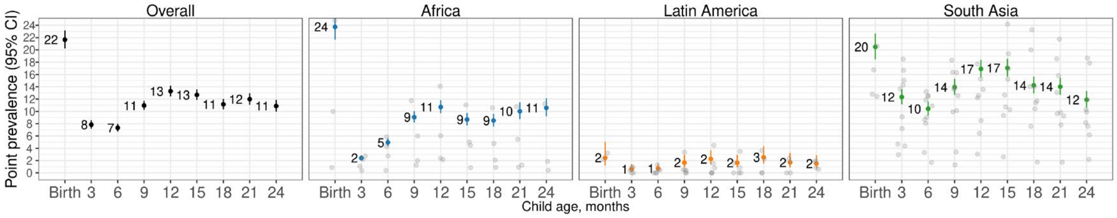

2.3 Age-specific incidence

2.3.1 Random effects

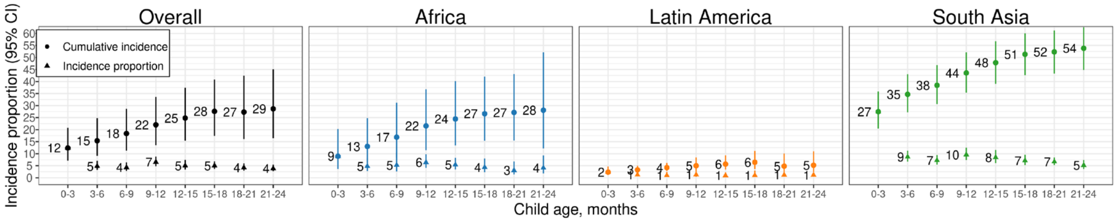

2.3.2 Fixed effects

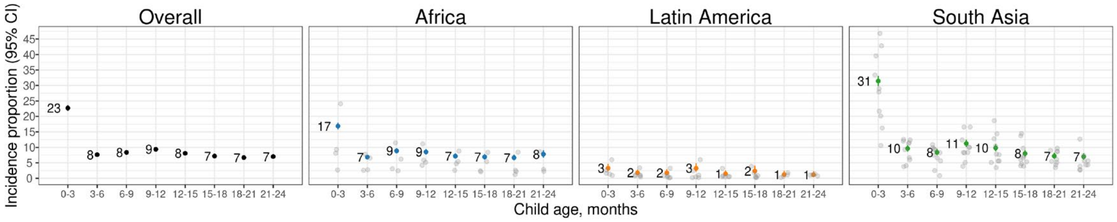

2.4 Age-specific incidence rate

2.4.1 Random effects

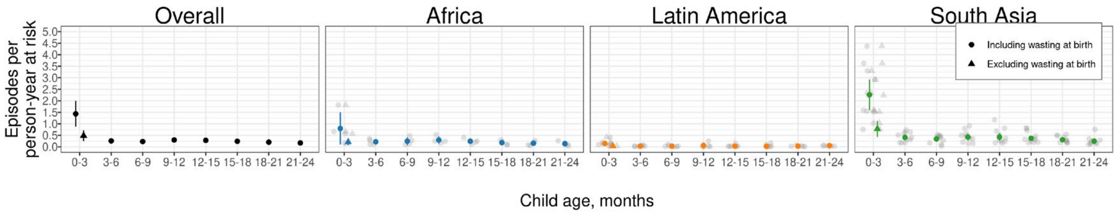

2.4.2 Fixed effects

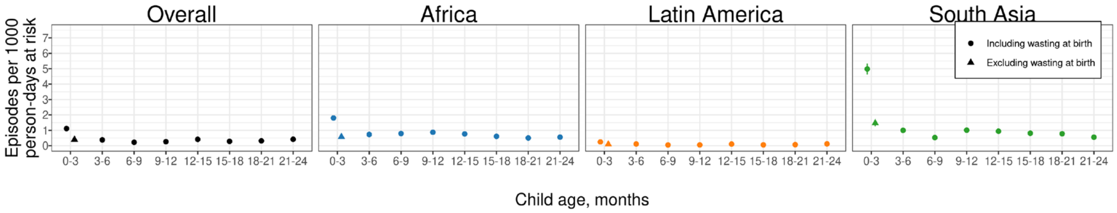

2.5 Age-specific recovery

2.5.1 Random effects

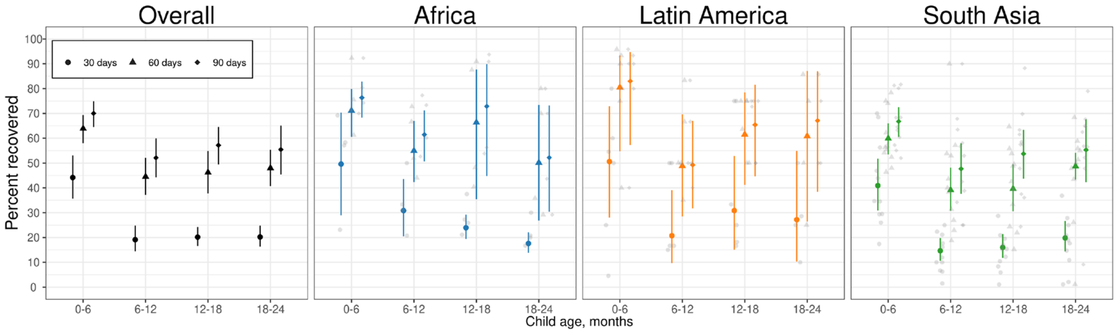

2.5.2 Fixed effects

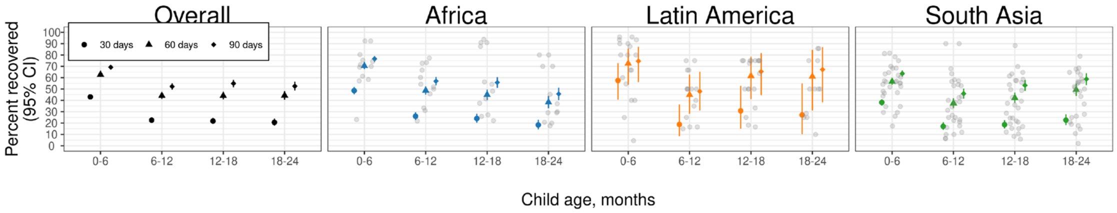

2.6 Age-specific prevalence of severe wasting

2.6.1 Random effects

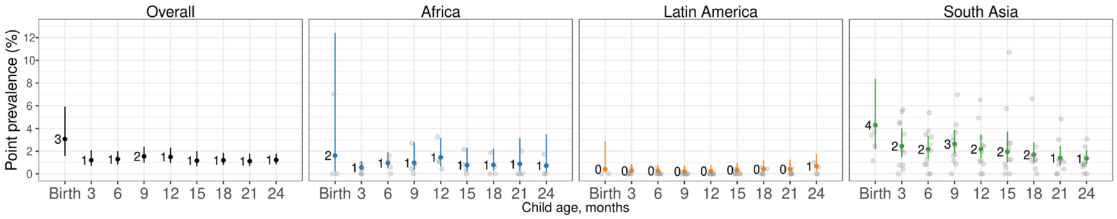

2.6.2 Fixed effects

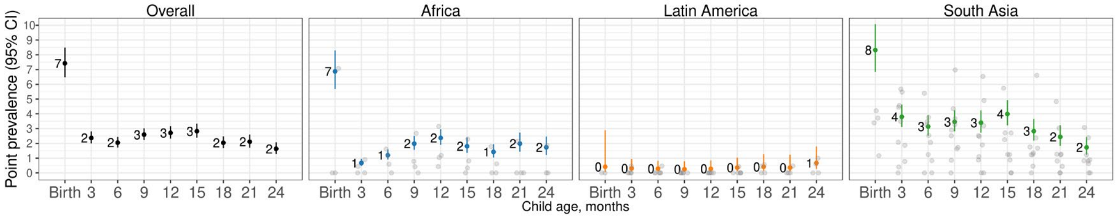

2.7 Age-specific longitudinal prevalence of persistent wasting

2.7.1 Random effects

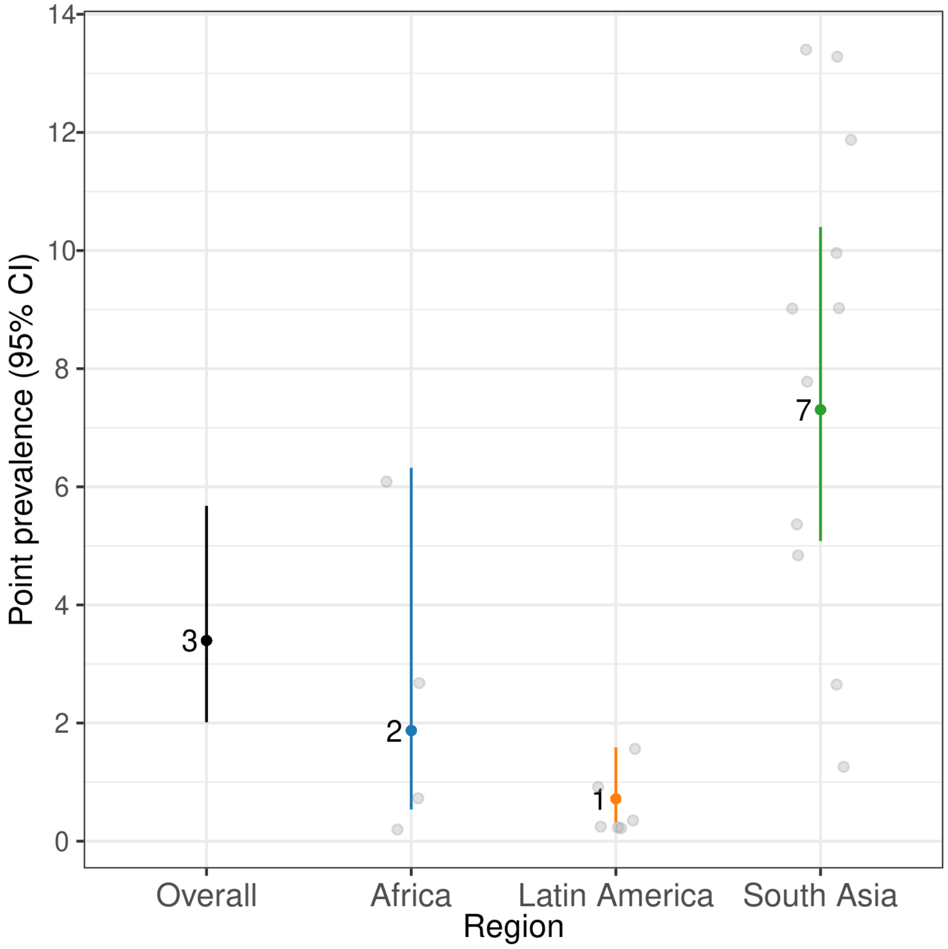

2.7.2 Fixed effects

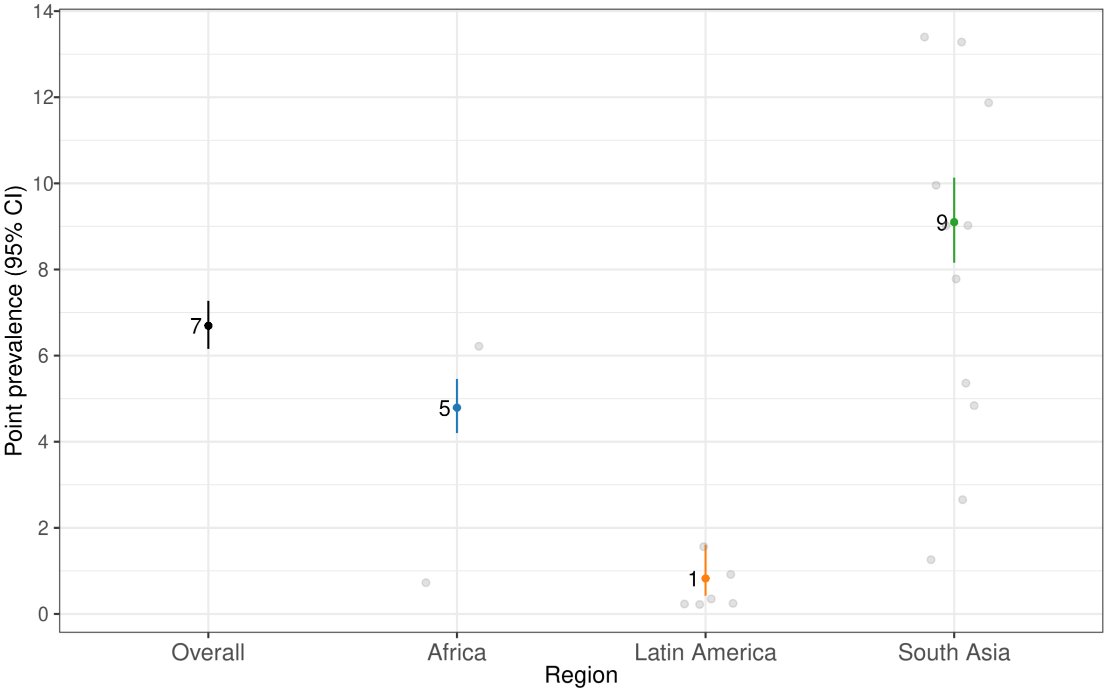

2.8 Age-specific prevalence of concurrent wasting and stunting

2.8.1 Random effects

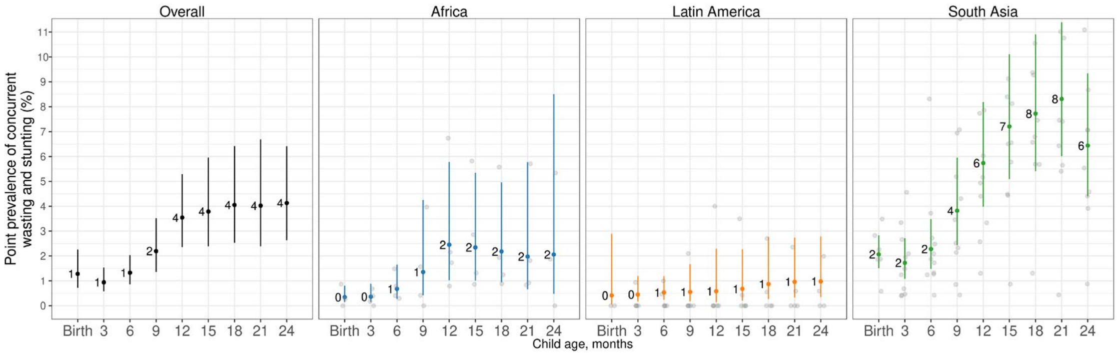

2.8.2 Fixed effects

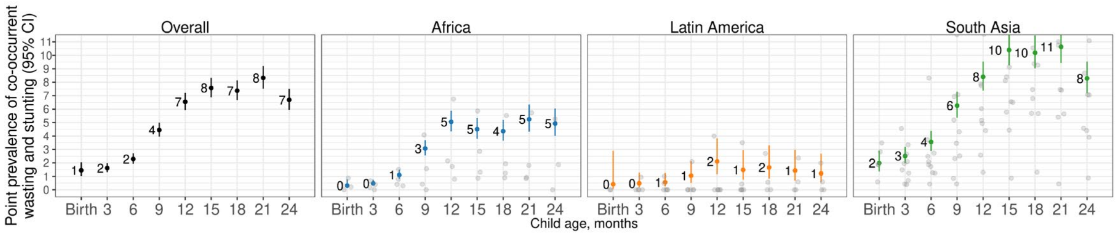

Supplementary Note 3. Sensitivity analysis comparing wasting defined via weight-for-length versus middle-upper arm circumference

3.1 Overview

Middle-upper arm circumference (MUAC) is an alternative anthropometry measure used to define wasting, but it was only measured in a subset of cohorts.

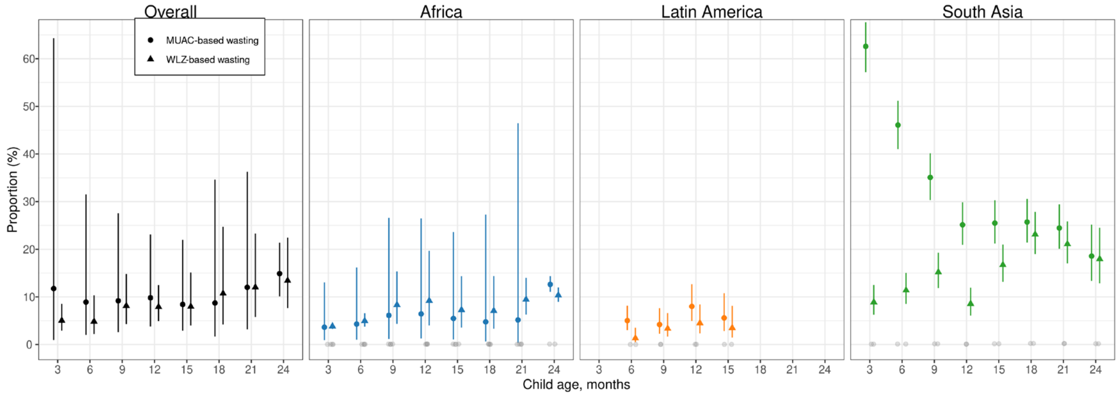

Supplementary Note 4. Sensitivity analysis dropping at-Birth measures in Kenaba

4.1 Overview

Here, we re-estimate primary results after dropping the observations of children at birth within the MRC Kenaba cohort, which used a different team to measure child anthropometry at birth from the trained anthropometrists used in follow-up measurements. While other cohort data from the Kenaba area show that children tend to experience decreased LAZ after birth, in the MRC Kenaba data used in this analysis, we see a high birth LAZ (approximately 0) and a rapid drop in LAZ in follow-up measurements (approximately -1 at one month). we see that the at birth measurements LAZ is ~0, and then for the follow-up measurements after the mean LAZ is ~ -1 (and the mean LAZ of just the subsequent follow-up visit at one month is ~ -1). We calculated 30% of measurements taken within two weeks of birth are lower than the at birth measurement beyond the technical error of measurement, and so are unrealistic decreases in child length, and 43% of follow-up measurements within two weeks of birth are less than the at-birth measurement by any amount.

4.2 Mean WLZ by region

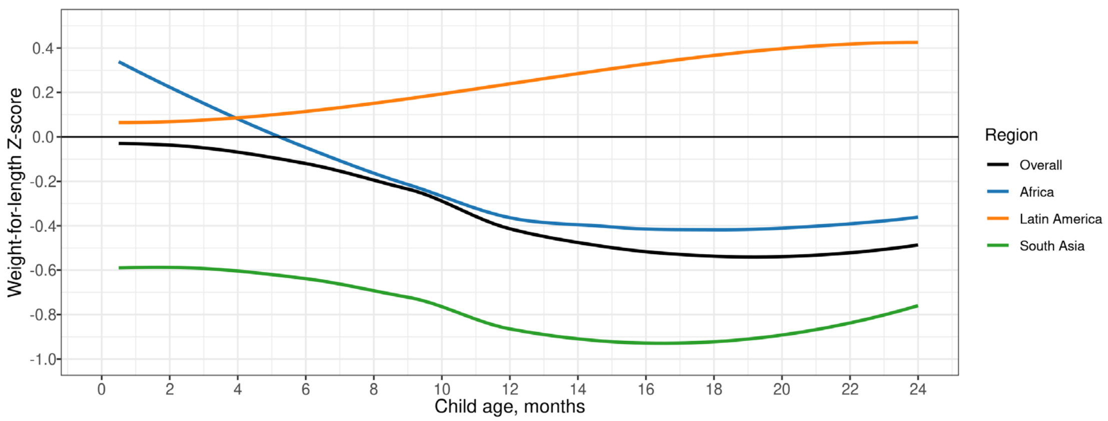

4.3 Age-specific prevalence

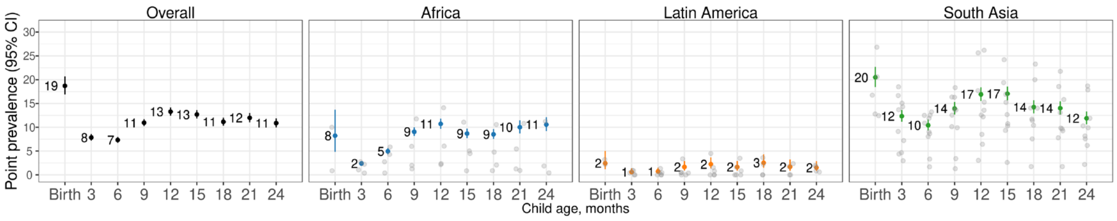

4.4 Age-specific incidence

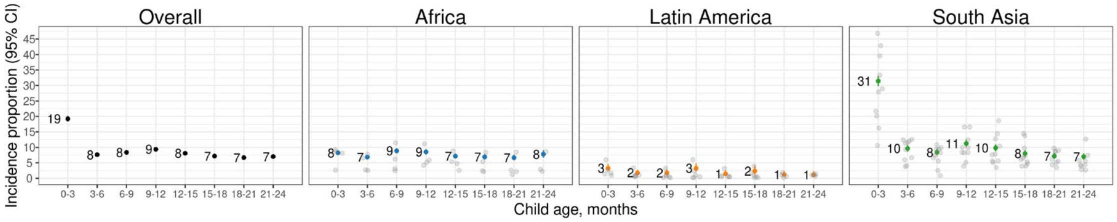

4.5 Age-specific incidence rate

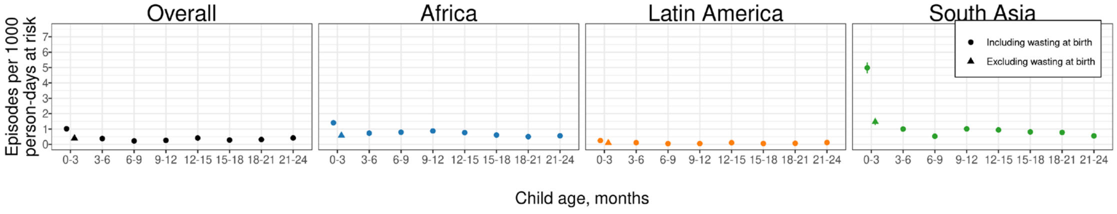

4.6 Age-specific recovery

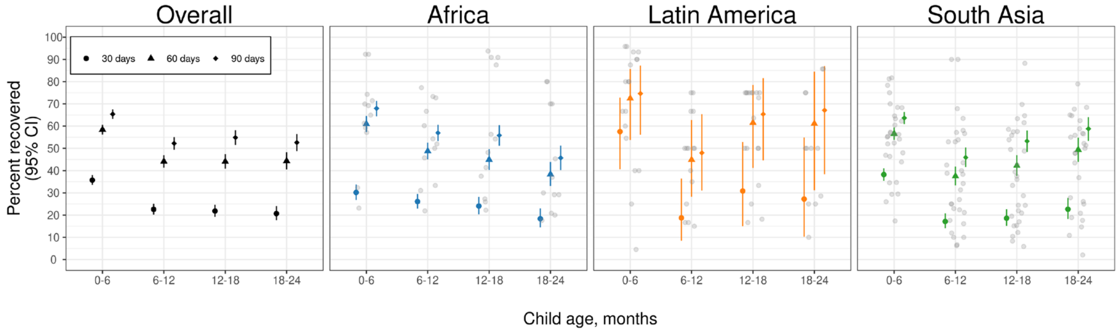

4.7 Age-specific prevalence of severe wasting

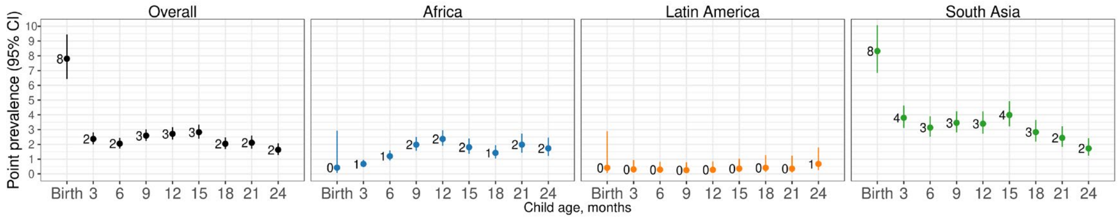

4.8 Age-specific longitudinal prevalence of persistent wasting

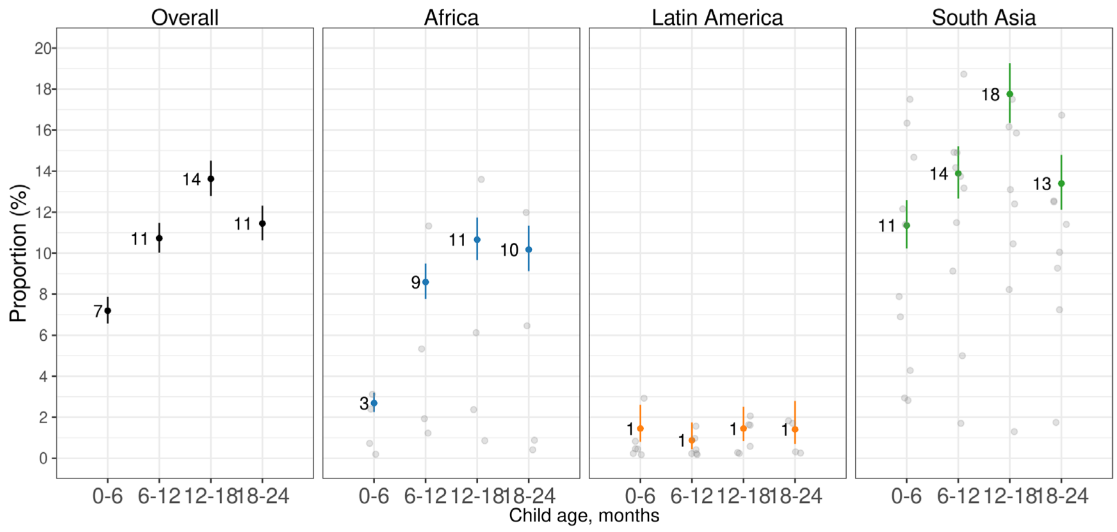

4.9 Age-specific prevalence of concurrent wasting and stunting

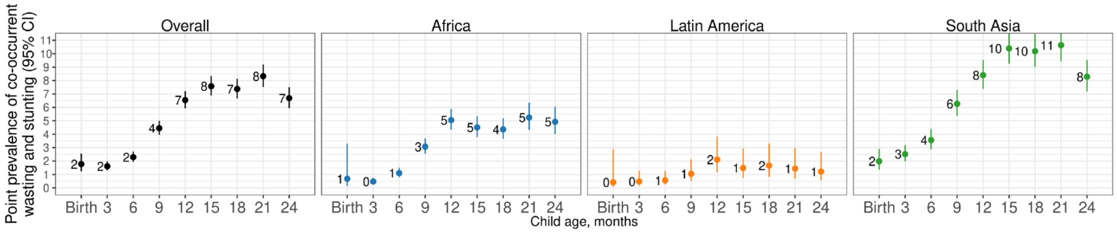

4.10 Age-specific prevalence of underweight (weight-for-age Z-score <-2)

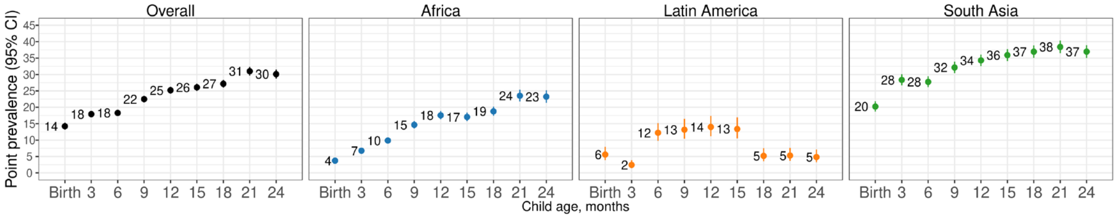

Supplementary Note 5. Incidence Rate Sensitivity

This figure examines the effect of wasting recovery definitions on age-specific incidence rates, and shows that incidence rates were relatively insensitive to the duration a child needed a WLZ > -2 to be considered recovered.

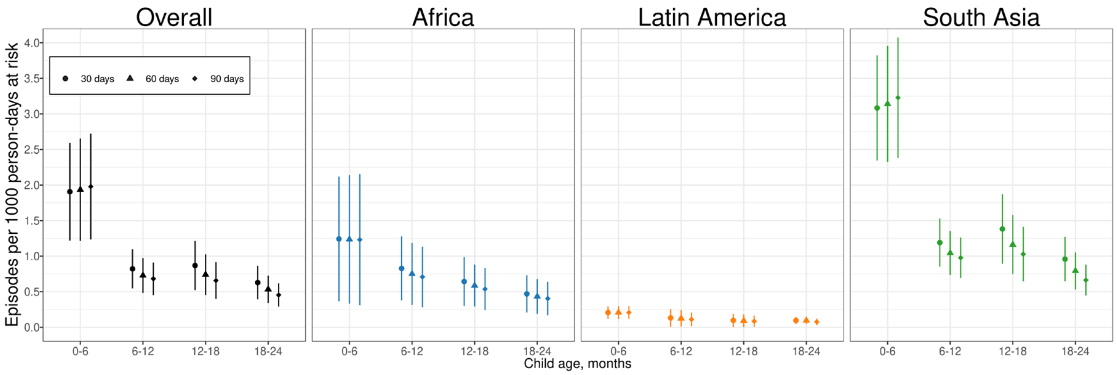

Supplementary Note 6. Underweight descriptive statistics

This chapter presents age-specific prevalence and incidence results for underweight (weight-for-age Z-score < -2) in the KI cohorts.

6.1 Weight-for-age Z-score (WAZ)

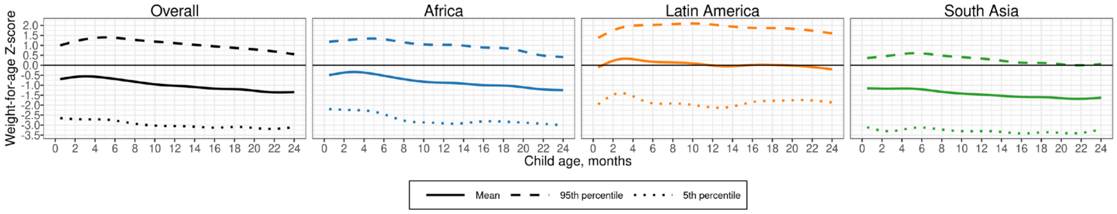

6.2 Age-specific prevalence

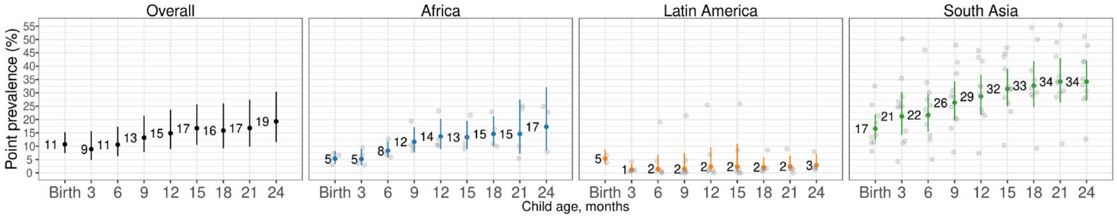

6.3 Age-specific incidence

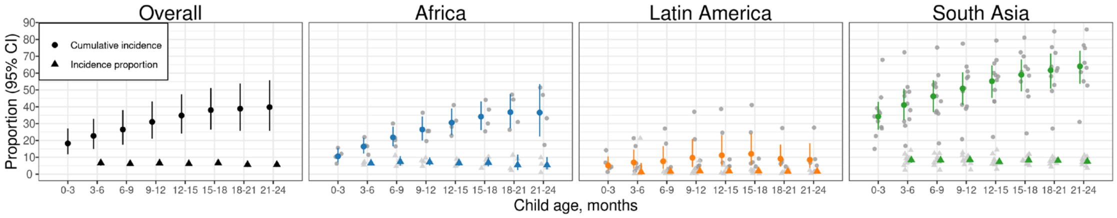

6.4 Age-specific incidence rate

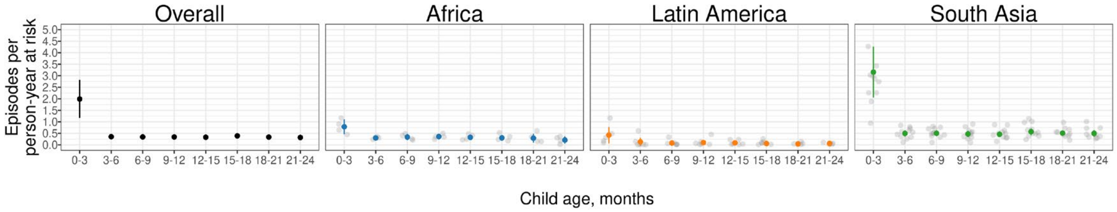

6.5 Age-specific recovery

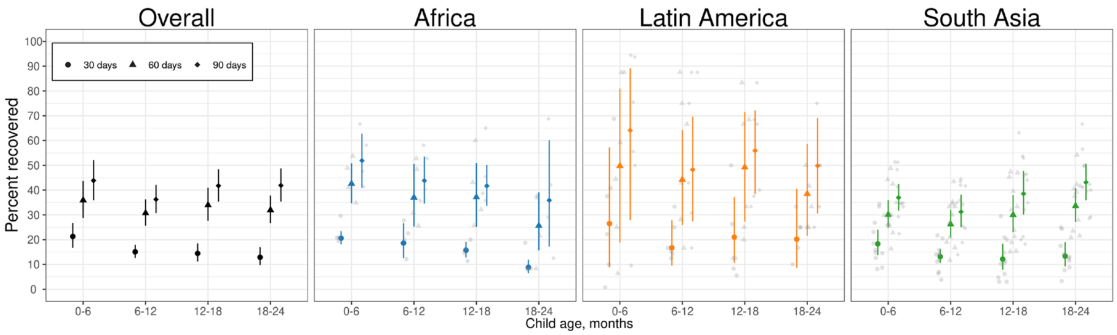

6.6 Age-specific prevalence of severe underweight

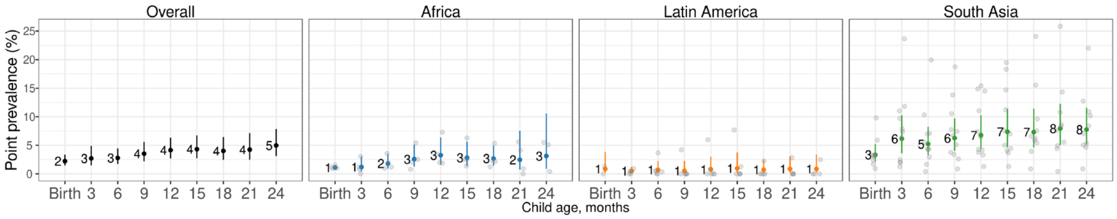

6.7 Age-specific longitudinal prevalence of persistent underweight

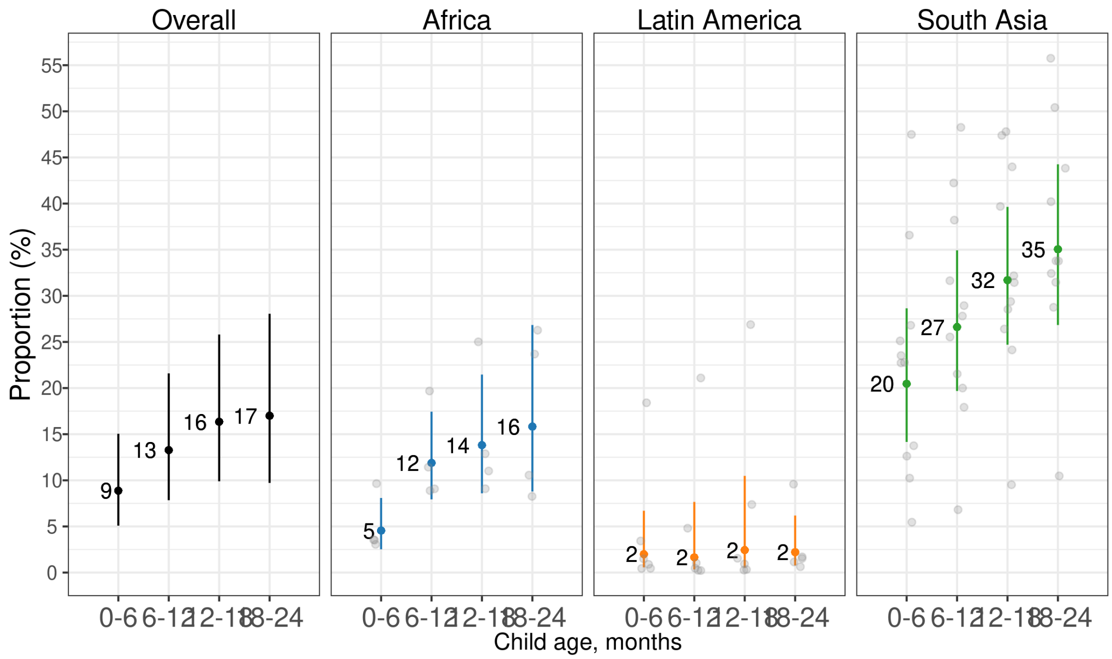

Supplementary Note 7. Prevalence and incidence of severe growth failure

7.1 Overview

This chapter presents age-specific prevalence and incidence results for severe wasting (weight-for-length Z-score < -3) and severe underweight (weight-for-age Z-score < -3) in the KI cohorts.

7.2 Age-specific prevalence of severe wasting

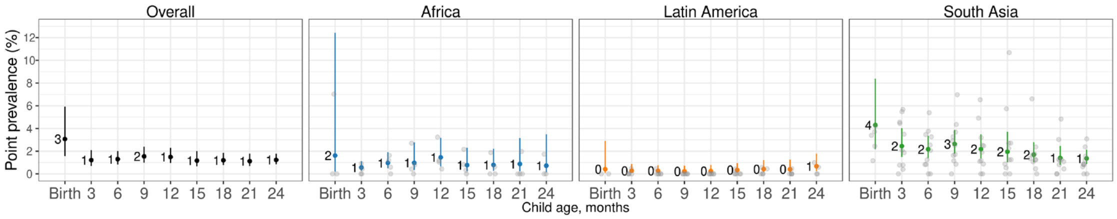

7.3 Age-specific cumulative incidence of severe wasting

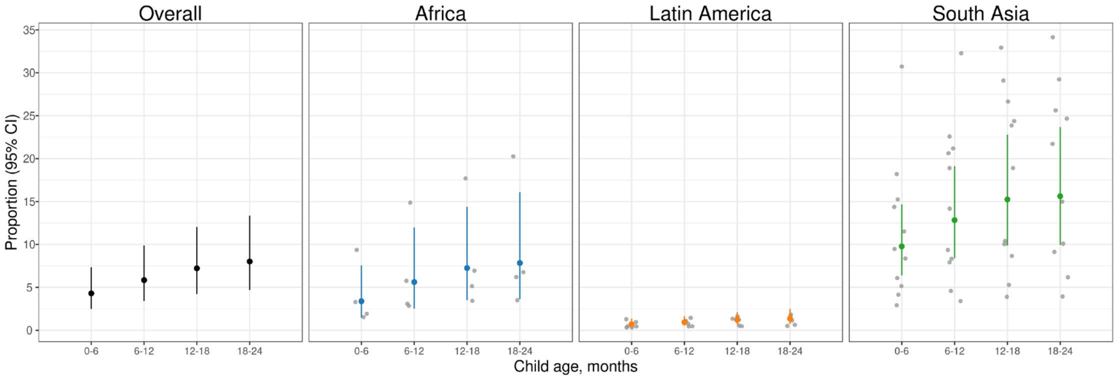

7.4 Age-specific prevalence of severe underweight

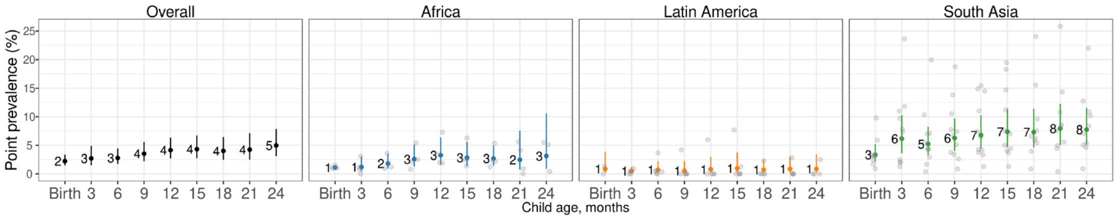

Supplementary Note 8. Cohort-specific estimates

8.1 Overview

Below are the cohort-specific estimates for the age-specific prevalences of wasting, severe wasting, persistent wasting, underweight, and concurrent wasting and stunting.

8.2 Age-specific prevalence

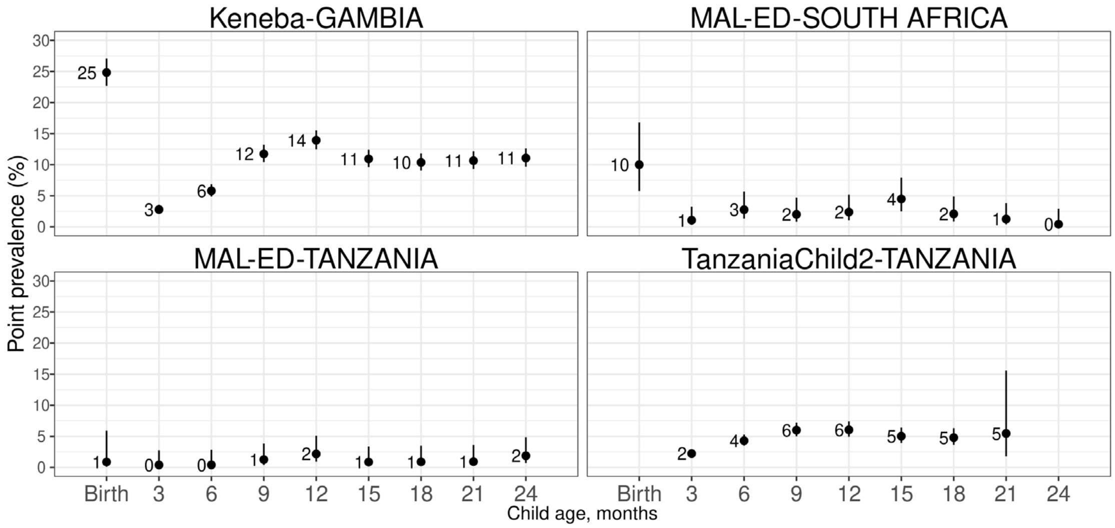

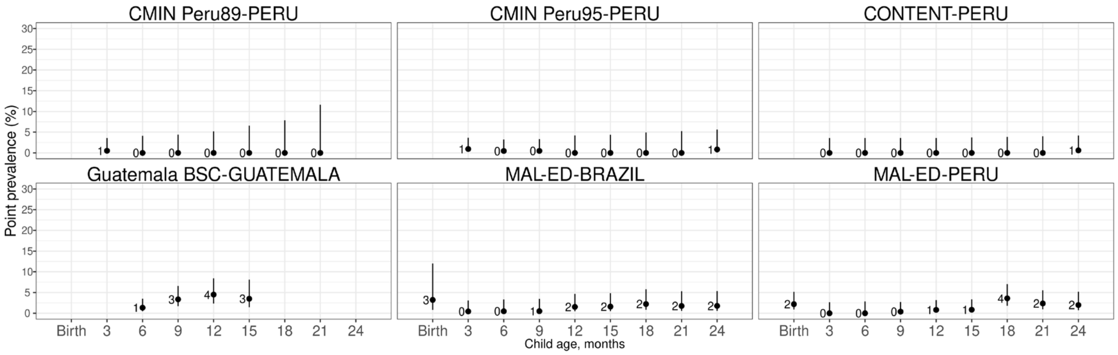

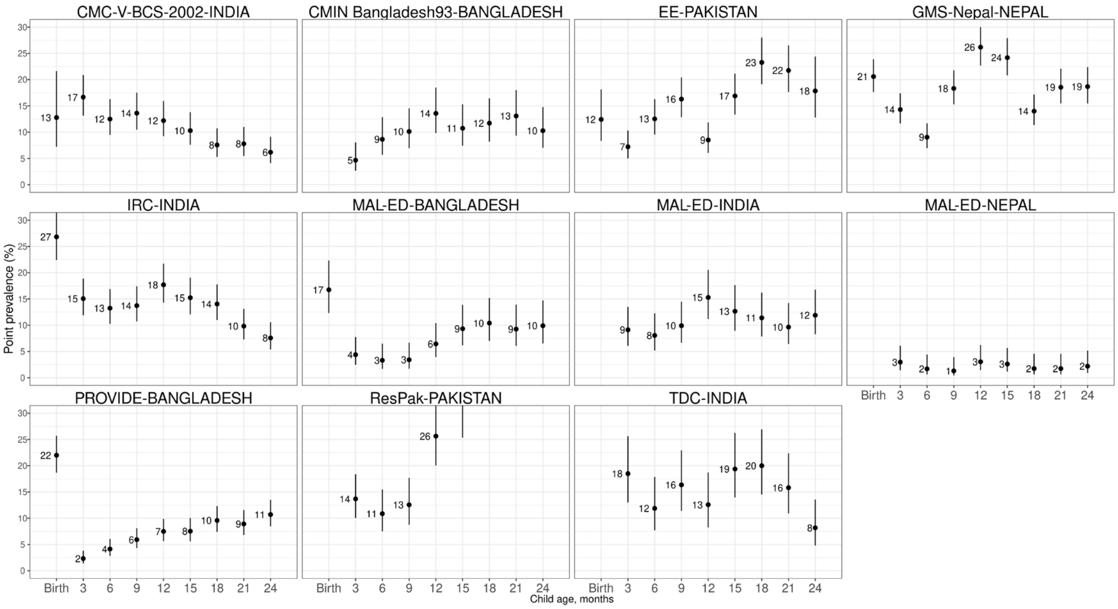

8.3 Age-specific prevalence of severe wasting

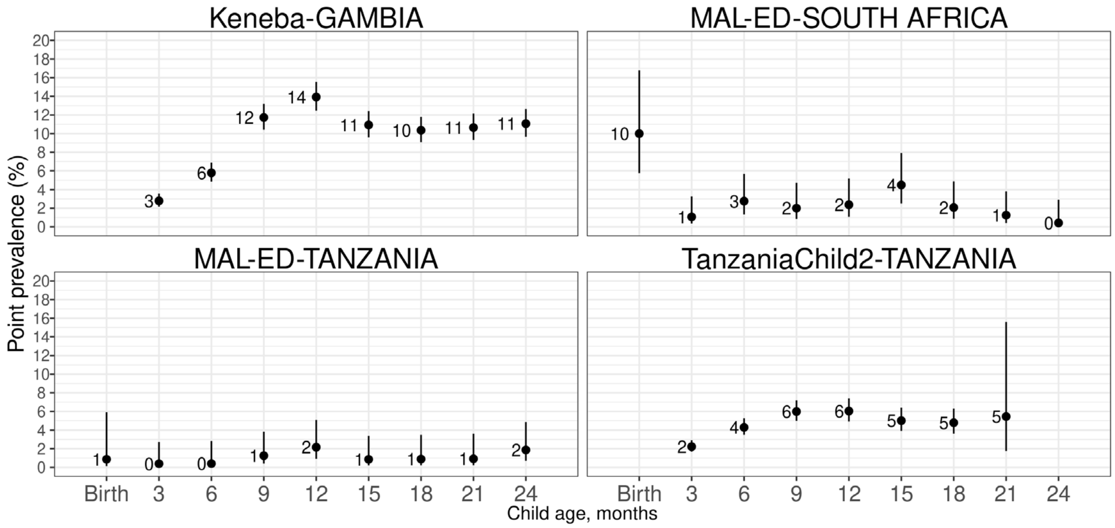

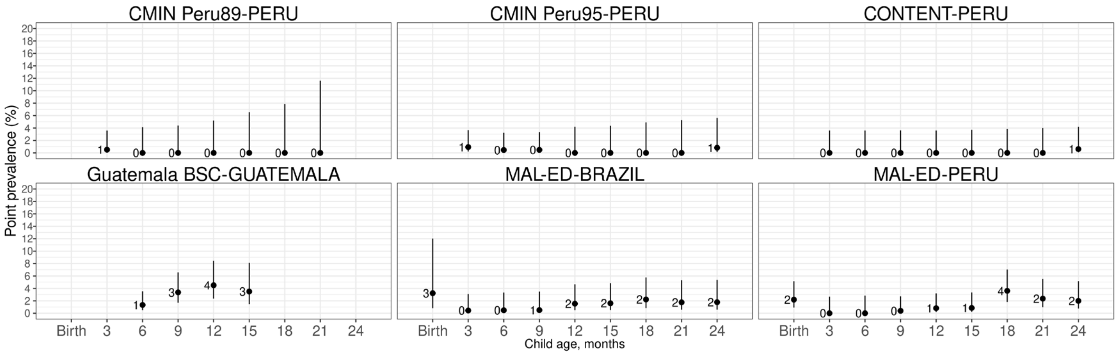

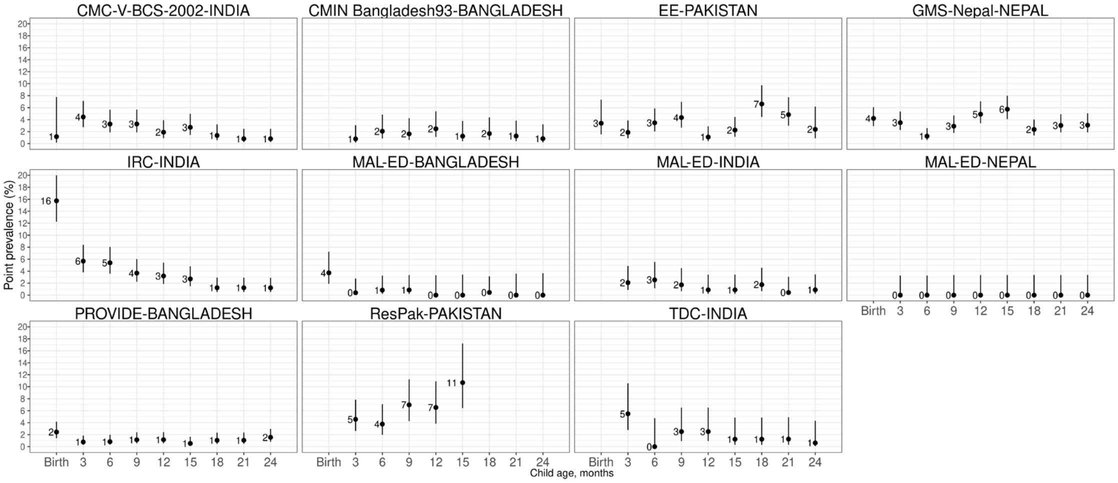

8.4 Age-specific longitudinal prevalence of persistent wasting

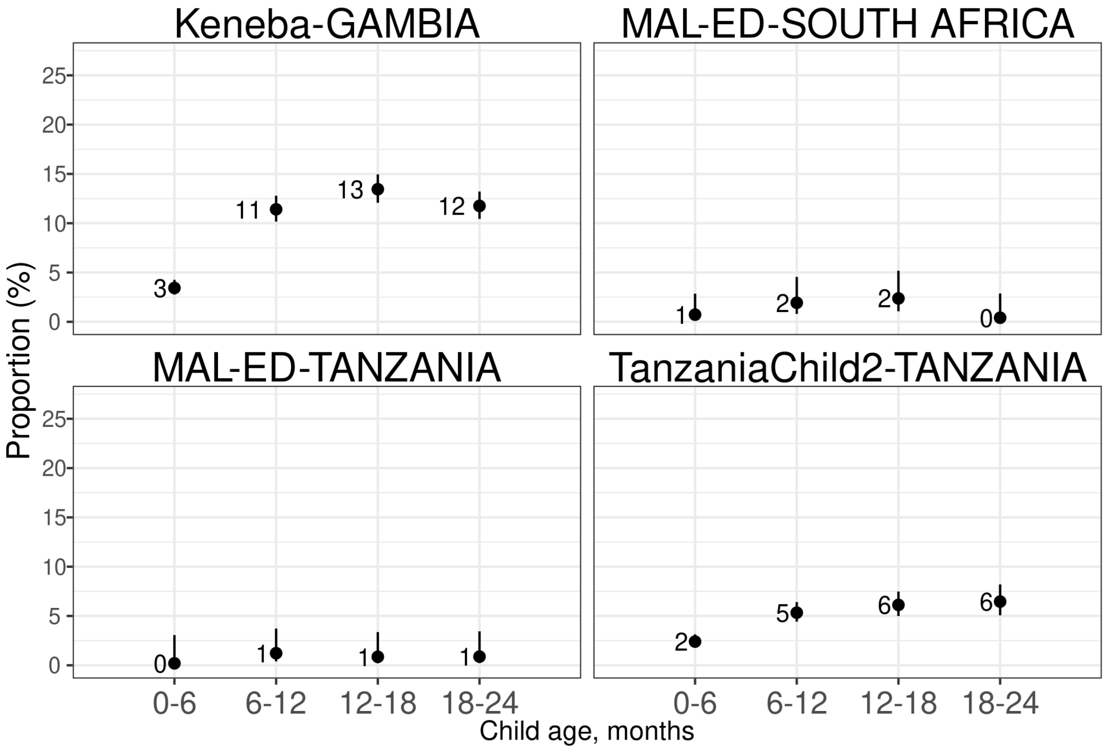

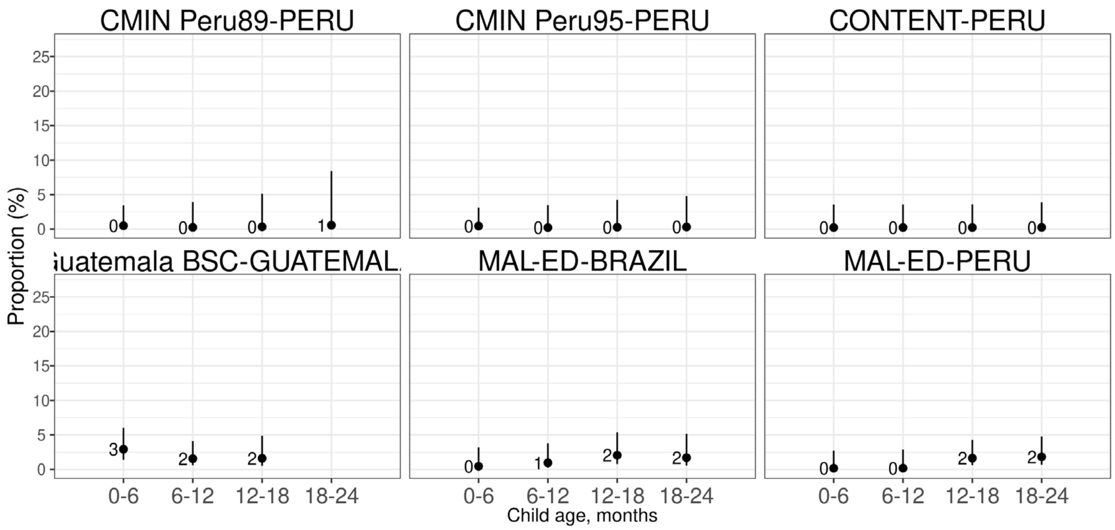
CMIN Peru89-PERU
CMIN Peru95-PERU
CONTENT-PERU
Guatemala BSC-GUATEMALA
MAL-ED-BRAZIL
MAL-ED-PERU
Proportion (%)
Child age, months
0-6
6-12
12-18
18-24

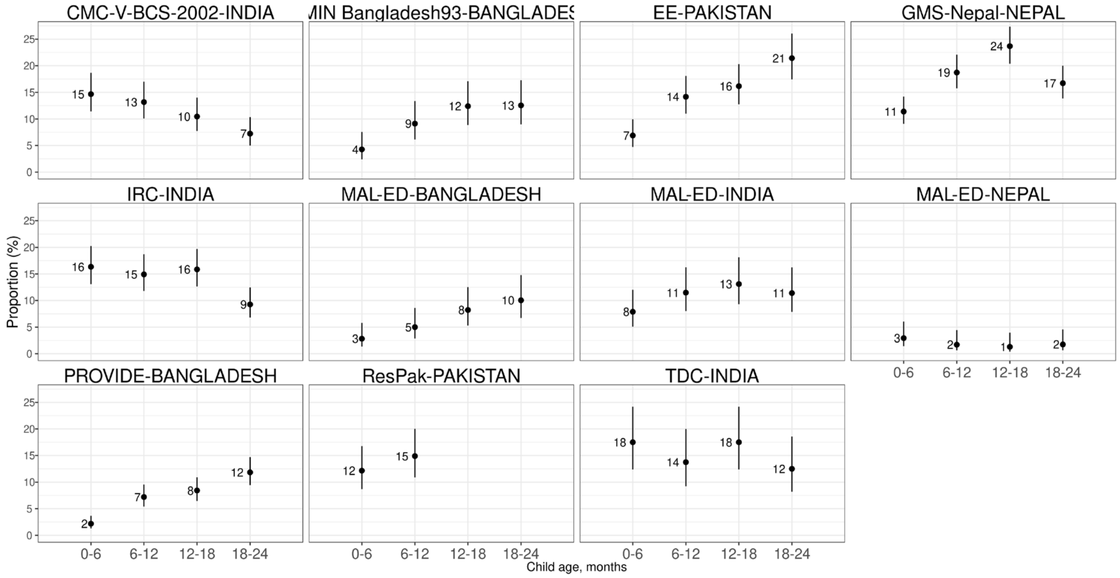
CMC-V-BCS-2002-INDIA
CMIN Bangladesh93-BANGLADESH
EE-PAKISTAN
GMS-Nepal-NEPAL
IRC-INDIA
MAL-ED-BANGLADESH
MAL-ED-INDIA
MAL-ED-NEPAL
PROVIDE-BANGLADESH
ResPak-PAKISTAN
TDC-INDIA
Proportion (%)
Child age, months
0-6
6-12
12-18
18-24

8.5 Age-specific prevalence of concurrent wasting and stunting

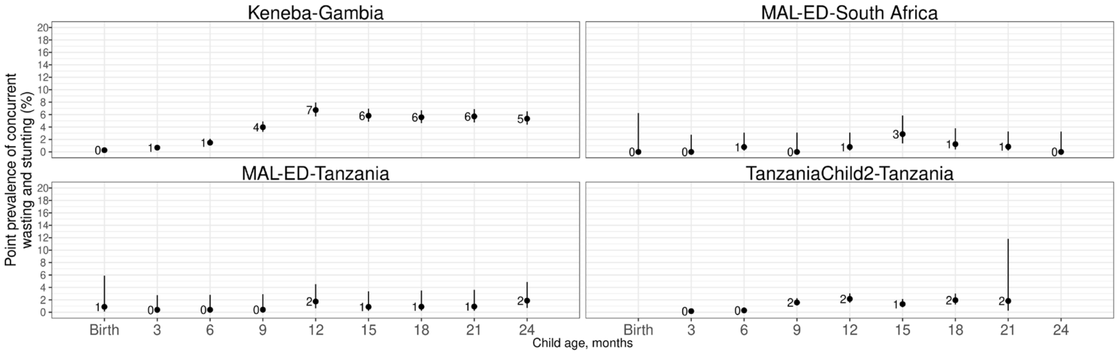

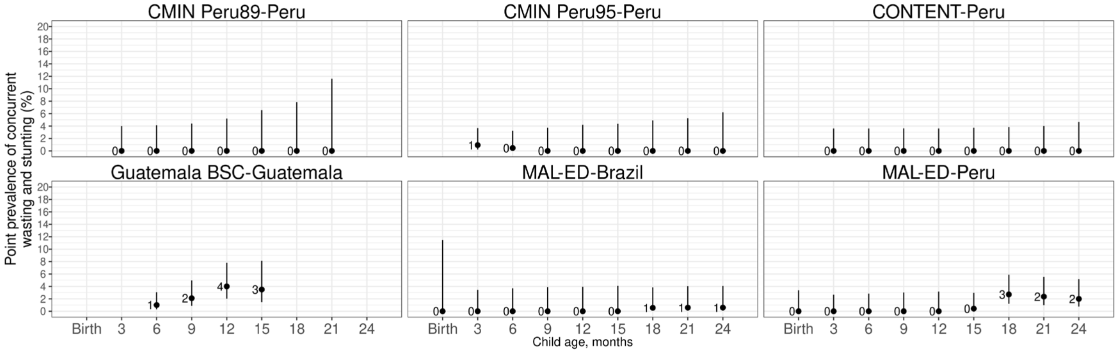

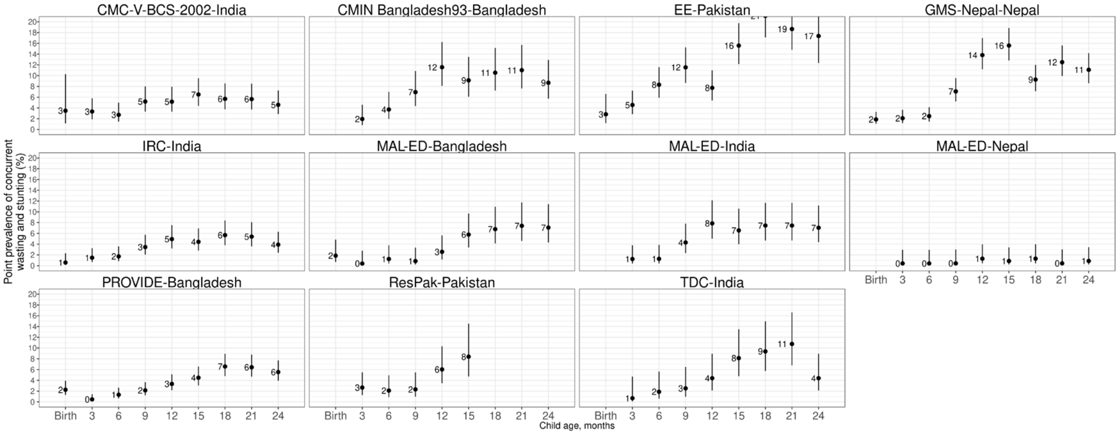

Supplement: Supplementary file 1 — This file contains Supplementary Notes 1–8. [file 41586_2023_6480_MOESM1_ESM.pdf]
